# The lung–muscle axis in COPD: a conceptual framework integrating evidence, mechanisms, and therapeutic perspectives

**DOI:** 10.3389/fimmu.2026.1907060

**Published:** 2026-09-08

**Authors:** Ganggeng Li, Dongmei Guan, Chongli Chen, Yanyun He, Wushuang Guo, Jie Li, Hongdan Wen, Zhitian Cheng, Hao Chen, Shuaichun Liu, Wenbin Wu

**Affiliations:** 1 Hospital of Chengdu University of Traditional Chinese Medicine, School of Clinical Medicine, Chengdu University of Traditional Chinese Medicine, Chengdu, China; 2 Wuwei Liangzhou Hospital, Wuwei, Gansu, China

**Keywords:** chronic obstructive pulmonary disease, lung–muscle axis, pulmonary rehabilitation, sarcopenia, skeletal muscle dysfunction, systemic inflammation

## Abstract

Skeletal muscle dysfunction (SMD) is a clinically important extrapulmonary manifestation of chronic obstructive pulmonary disease (COPD) and is associated with impaired exercise capacity, reduced physical function, increased exacerbation risk, and poorer clinical outcomes. Although traditionally viewed as a consequence of pulmonary disease progression, accumulating evidence suggests that pulmonary impairment and skeletal muscle abnormalities may reflect partially overlapping processes within the systemic pathobiology of COPD. This narrative review synthesizes clinical, imaging, genetic, mechanistic, and therapeutic evidence and uses the lung–muscle axis as a conceptual framework to organize established clinical relationships, shared systemic mechanisms, functional coupling, and candidate inter-organ signaling pathways. Available studies indicate that pulmonary abnormalities and SMD are associated and may share determinants including chronic inflammation, oxidative stress, mitochondrial dysfunction, metabolic dysregulation, cellular aging, and impaired tissue adaptation. Functional coupling among respiratory muscle performance, peripheral muscle capacity, and exercise tolerance further supports the clinical relevance of this framework. Circulating mediators, myokines, and extracellular vesicles have been proposed as potential mechanisms of inter-organ signaling; however, direct molecular communication and bidirectional causal effects remain incompletely established. These relationships vary according to disease phenotype, environmental exposure, disease stage, and host susceptibility. Clinically, the lung–muscle framework may broaden COPD phenotyping and management by complementing pulmonary assessment with measures of muscle function, physical performance, nutritional status, and imaging-derived muscle characteristics. Future longitudinal, multi-omics, and tissue-resolved studies are needed to clarify causal pathways, distinguish biologically relevant mediators from disease-associated biomarkers, and determine whether targeted interventions can preserve both respiratory and skeletal muscle function.

## 1 Introduction

Chronic obstructive pulmonary disease (COPD) is a heterogeneous chronic respiratory disorder characterized by persistent airflow limitation, respiratory symptoms, and variable clinical trajectories (1). Although COPD primarily affects the airways and lung parenchyma, it is increasingly recognized as a disease with important systemic consequences, including persistent inflammatory and immune dysregulation and extrapulmonary manifestations such as skeletal muscle dysfunction, cardiovascular comorbidities, metabolic abnormalities, and frailty (1, 2). These manifestations are associated with functional decline, poorer quality of life, and adverse clinical outcomes that are not fully captured by conventional pulmonary measurements.

Skeletal muscle dysfunction (SMD) is a clinically relevant extrapulmonary manifestation of COPD and an umbrella term encompassing abnormalities in muscle strength, endurance, quality, metabolism, and structure (3–5). Within this spectrum, clinically defined sarcopenia, muscle wasting, reduced muscle mass, and impaired physical performance represent related but non-equivalent constructs. These abnormalities have been associated with reduced exercise capacity, increased exacerbation risk, poorer quality of life, and higher mortality in COPD (6–9). Their manifestations also vary across muscle compartments because respiratory, locomotor, and trunk muscles are exposed to different mechanical and systemic stresses. The diaphragm is particularly influenced by chronic hyperinflation and altered mechanical loading, whereas peripheral locomotor muscles, including the quadriceps, are commonly affected by hypoxemia, systemic inflammation, physical inactivity, and metabolic disturbances (3, 10, 11). Computed tomography-derived measurements of the pectoral and paraspinal muscles provide complementary information on muscle reserve and composition (12, 13). Accordingly, COPD-associated muscle impairment cannot be adequately characterized by a single phenotype or assessment modality.

Longitudinal studies have associated reduced muscle strength, altered body composition, and impaired physical performance with subsequent pulmonary outcomes and prognostic information beyond conventional lung function measures (14, 15). Together with evidence linking pulmonary disease burden to skeletal muscle impairment, these observations support a broader perspective in which pulmonary and muscular abnormalities may share biological determinants and become functionally coupled during COPD progression (4, 16). However, these findings do not establish direct bidirectional signaling between pulmonary and skeletal muscle compartments.

In this review, the “lung–muscle axis” is used as a hierarchical conceptual framework for organizing the biological processes and functional relationships linking pulmonary pathology and skeletal muscle dysfunction in COPD. It does not denote a single linear pathway or a universally established bidirectional causal mechanism. The framework comprises five domains: (1) shared systemic determinants, including aging, smoking exposure, physical inactivity, nutritional imbalance, and chronic inflammation, that may independently affect pulmonary and skeletal muscle compartments (17); (2) lung-related effects on skeletal muscle homeostasis involving hypoxemia, inflammatory activation, oxidative stress, and metabolic disturbances (18); (3) potential muscle-related influences on respiratory symptoms, ventilatory demand, and functional reserve (19, 20); (4) functional and biomechanical coupling among respiratory muscle performance, peripheral muscle capacity, and exercise tolerance (4, 16); and (5) candidate molecular signaling pathways involving circulating mediators, myokines, and extracellular vesicles (21–23).

These domains are complementary but represent distinct biological processes and levels of evidence. Shared systemic determinants may affect pulmonary and skeletal muscle compartments concurrently without requiring direct inter-organ signaling, whereas associations between muscle impairment and respiratory outcomes may reflect common disease burden, reduced physiological reserve, or functional coupling. Clinical studies provide consistent evidence for relationships among pulmonary disease burden, skeletal muscle abnormalities, and functional outcomes, but direct molecular signaling between lung and muscle remains insufficiently validated. The framework is therefore used to distinguish established clinical relationships from biologically plausible but incompletely tested mechanisms. Previous reviews have provided important insights into the mechanisms of COPD-associated skeletal muscle dysfunction. Building on this work, the present review integrates clinical, genetic, imaging, mechanistic, and therapeutic evidence to provide a broader perspective on lung–muscle relationships in COPD and to identify remaining uncertainties and priorities for future research.

## 2 Methods: literature identification and evidence framework

This narrative review synthesized evidence on the relationships between pulmonary pathology and skeletal muscle dysfunction in COPD. PubMed, Web of Science, and Scopus were searched from database inception to June 30, 2026. Additional relevant studies identified through reference-list screening and during manuscript revision were considered where appropriate. The search strategy combined terms related to COPD (“chronic obstructive pulmonary disease”, “emphysema”, and “airflow limitation”), skeletal muscle alterations (“skeletal muscle dysfunction”, “sarcopenia”, “muscle wasting”, and “respiratory muscle”), and potential inter-organ communication (“lung–muscle axis”, “organ crosstalk”, “systemic inflammation”, “extracellular vesicles”, and “myokines”), with no language restrictions. Studies were eligible if they examined clinical associations, longitudinal outcomes, imaging-based assessments, genetic evidence, biological mechanisms, or therapeutic interventions relevant to pulmonary and skeletal muscle phenotypes in COPD. Titles and abstracts were independently screened by two reviewers, followed by full-text assessment of potentially eligible studies. Disagreements regarding eligibility or interpretation were resolved through discussion and consensus. Data were extracted on study design, population characteristics, muscle-related constructs, pulmonary phenotypes, the direction and magnitude of reported associations where applicable, principal findings, and the source and context of evidence. Evidence from non-COPD populations or experimental systems was included only when it provided mechanistic insights relevant to COPD biology and was interpreted as supportive, rather than direct, evidence of COPD-associated lung–muscle relationships. Because this was a narrative review, no quantitative meta-analysis or formal risk-of-bias instrument was applied. Instead, studies were qualitatively synthesized and appraised according to study design, population relevance, consistency of findings, experimental validation, biological relevance, and directness of evidence. Evidence was categorized as deriving from human longitudinal or cross-sectional studies, randomized clinical trials, Mendelian randomization studies, COPD-specific experimental models, non-COPD mechanistic investigations, or hypothesis-generating studies.

## 3 Genetic evidence informing lung–muscle relationships

Mendelian randomization (MR) studies have examined potential causal relationships between muscle-related traits and pulmonary phenotypes. However, these analyses primarily evaluate genetically predicted quantitative traits, including appendicular lean mass, handgrip strength, walking pace, and fat-free mass. Each trait captures a specific dimension of muscle biology and therefore provides only a partial representation of the broader spectrum of skeletal muscle dysfunction.

Several MR studies have reported genetically informed associations between muscle-related traits and COPD or pulmonary function (**Table 1**). Fu and Yang conducted bidirectional MR analyses and found that genetically predicted appendicular lean mass was associated with a lower risk of COPD, whereas the reverse analysis did not support an effect of COPD liability on appendicular lean mass (24). Similarly, genetically predicted handgrip strength has been associated with higher forced expiratory volume in one second (FEV_1_) and forced vital capacity (FVC), but not with the FEV_1_/FVC ratio (25). Other MR studies examining walking pace and additional muscle-related quantitative traits have identified potential relationships with COPD susceptibility, although the direction, magnitude, and robustness of these associations vary across traits and analytical models (26–28). Collectively, these findings provide genetically informed evidence consistent with potential causal relationships between selected muscle-related traits and pulmonary phenotypes, but they do not establish generalized bidirectional causality or direct biological communication between the lung and skeletal muscle. Further mechanistic and longitudinal studies are required to determine the biological pathways underlying these associations.

**Table 1:** Mendelian randomization evidence for muscle–pulmonary relationships.

Direction tested	Key finding	Interpretation	Reference
ALM ↔ COPD; ALM ↔ FEV_1_/FVC	Higher ALM was associated with lower COPD risk; reverse and FEV_1_/FVC associations were null.	Unidirectional ALM→COPD evidence.	(24)
Walking pace and HGS ↔ COPD	Walking pace was associated with COPD in both directions; other findings relied on relaxed-threshold instruments.	Suggestive bidirectional evidence limited to walking pace.	(27)
Muscle-related traits ↔ COPD	COPD → left-HGS and walking pace → COPD were identified but attenuated after multivariable adjustment.	Different traits were implicated in each direction; bidirectionality was not established.	(26)
HGS, walking speed and fat-free mass → COPD	All three traits showed nominal associations with COPD; reverse effects were not tested.	One-directional, trait-specific evidence.	(28)
HGS → pulmonary function	Higher HGS was associated with higher FVC and FEV_1_, but not FEV_1_/FVC.	Pulmonary-function evidence only.	(25)

ALM, appendicular lean mass; COPD, chronic obstructive pulmonary disease; FEV_1_, forced expiratory volume in one second; FVC, forced vital capacity; HGS, handgrip strength; MR, Mendelian randomization.

## 4 Clinical evidence informing the lung–muscle framework

Clinical studies consistently report associations among pulmonary impairment, skeletal muscle dysfunction, functional limitation, and adverse outcomes in COPD. Skeletal muscle abnormalities are associated with greater symptom burden, reduced physical capacity, increased exacerbation risk, and poorer prognosis (**Table 2**). However, the available clinical evidence primarily supports phenotypic associations and functional coupling; direct bidirectional biological communication between pulmonary and skeletal muscle compartments has not been established.

**Table 2:** Clinical evidence linking pulmonary and skeletal muscle phenotypes in COPD.

Domain	Core observation	Evidence base	References
Prevalence and prognosis	Sarcopenia and muscle loss; exacerbation, hospitalization, and mortality risk	Systematic reviews; longitudinal cohorts	(6–9, 29)
Pulmonary–muscle coupling	Pulmonary outcomes linked to muscle measures; respiratory weakness and quadriceps dysfunction	Longitudinal cohorts; physiological studies	(4, 14, 30–33)
Functional phenotyping	Complementary assessment of mass, strength, endurance, performance, and respiratory muscle function	Clinical and prognostic studies	(34–37)
Stage-related variation	Muscle impairment in stable disease; accelerated deterioration during exacerbations	Observational studies; randomized trial	(38–44)

Evidence levels reflect the strength and context of available data and do not imply equivalent evidence for direct bidirectional organ communication. COPD, chronic obstructive pulmonary disease.

### 4.1 Prevalence and prognostic burden

Skeletal muscle dysfunction is common in COPD and has substantial prognostic relevance. The reported prevalence of sarcopenia varies according to patient characteristics, disease severity, diagnostic criteria, and assessment methods; nevertheless, sarcopenia is consistently associated with adverse clinical outcomes (6). Patients with COPD and sarcopenia have increased risks of acute exacerbations, pneumonia, and all-cause mortality, while progressive muscle loss is associated with poorer long-term outcomes (7, 29). Large cohort studies further indicate that muscle loss-related phenotypes are associated with higher in-hospital mortality, longer hospital stays, and greater healthcare utilization (9). These findings support skeletal muscle impairment as an important component of clinical risk stratification in COPD.

### 4.2 Lung–muscle associations and functional coupling

Epidemiological and longitudinal studies have reported associations between reduced muscle-related measures and subsequent pulmonary outcomes, although the direction and magnitude of these relationships vary across populations and measured traits (14, 30, 31). Conversely, COPD-related physiological stressors, including chronic hypoxemia, physical inactivity, and systemic inflammation, may impair skeletal muscle homeostasis (4). At the functional level, reduced inspiratory and expiratory muscle strength is associated with greater airflow limitation, exercise intolerance, and dyspnea severity (32). Peripheral muscle dysfunction, particularly reduced quadriceps endurance, may also occur early in the disease and is not necessarily proportional to the loss of muscle strength (33). This dissociation suggests that muscle strength, endurance, and functional performance capture distinct dimensions of COPD-associated muscle impairment. Respiratory and peripheral muscle abnormalities should therefore be assessed as complementary, but non-interchangeable, clinical phenotypes.

### 4.3 Functional stratification and treatable traits

Clinical assessment of COPD-associated muscle impairment should extend beyond muscle mass to include strength, endurance, physical performance, and exercise capacity. The 1-minute sit-to-stand test (1STST) and six-minute walk distance (6MWD) provide complementary measures of physical performance and exercise capacity in COPD (34, 35). Skeletal muscle dysfunction has therefore been proposed as a clinically relevant treatable trait in COPD. Reduced muscle mass and impaired physical performance are associated with poorer functional trajectories and quality-of-life outcomes (36). Assessment of respiratory muscle strength alongside conventional indices, including FEV_1_ and peak oxygen uptake, may further improve prognostic evaluation (37). Respiratory muscle strength, endurance, activation, excursion, and ultrasound-derived parameters represent distinct physiological domains and should not be interpreted as equivalent measures.

### 4.4 Stage-dependent dynamics

The relationship between pulmonary disease burden and skeletal muscle impairment may vary across the clinical course of COPD. In stable disease, systemic inflammation, metabolic abnormalities, hypoxemia, and physical inactivity are implicated in muscle impairment, which may be detectable even in patients with relatively mild airflow limitation (38, 39).

Acute exacerbations are associated with accelerated deterioration in muscle function. During hospitalization, muscle strength may decline rapidly in parallel with inflammatory activation and impaired anabolic balance (40). Recurrent exacerbations are also associated with reduced physical activity, lower exercise capacity, and persistent functional impairment, potentially increasing cumulative muscle loss over time (41, 42). Recovery is frequently incomplete, and persistent impairment in muscle function is associated with poorer subsequent outcomes and higher mortality risk (43). Rehabilitation and resistance training may support functional recovery after exacerbations, although the optimal timing, intensity, and durability of benefit remain uncertain (44). These findings support the inclusion of skeletal muscle phenotyping in multidimensional COPD assessment.

## 5 Multimodal imaging evidence informing the lung–muscle framework

Multimodal imaging enables concurrent characterization of pulmonary abnormalities and skeletal muscle phenotypes across structural, functional, and prognostic domains (**Table 3**). By quantifying both compartments within the same clinical setting, these techniques can identify coexisting patterns of pulmonary disease and muscle impairment. Imaging findings should nevertheless be interpreted primarily as phenotypic associations, because most available studies are cross-sectional and do not establish temporal coordination or causal coupling between pulmonary and skeletal muscle changes.

**Table 3:** Multimodal imaging assessment of COPD-associated muscle phenotypes.

Phenotype	Imaging metric	Clinical correlate	Evidence	References
Muscle quantity	CT-derived erector spinae and pectoralis area; MRI-derived muscle area and volume	Disease severity, muscle mass, and prognosis	Cross-sectional, prognostic, and methodological studies	(13, 45, 46, 55)
Muscle composition	CT attenuation	Myosteatosis, physical activity, and exercise capacity	Observational study	(48)
Diaphragm morphology and function	Ultrasound-derived thickness, thickening, excursion, and force reserve; MRI functional measures	Respiratory reserve, pulmonary function, noninvasive ventilation outcomes, and exacerbation risk	Prospective observational cohorts	(49–51, 56)
Peripheral muscle morphology	Ultrasound-derived rectus femoris thickness and cross-sectional area	Fat-free mass, quadriceps strength, and exercise tolerance	Cross-sectional and exploratory studies	(52, 53)
Muscle mechanical properties	Shear-wave elastography	Muscle stiffness and lower-limb dysfunction	Exploratory study	(54)

CT, computed tomography; MRI, magnetic resonance imaging.

### 5.1 Computed tomography

Quantitative chest computed tomography (CT) permits simultaneous assessment of pulmonary structural abnormalities and selected trunk muscles. Measurements of the erector spinae and pectoral muscles have been associated with COPD severity and adverse clinical outcomes (13, 45–47). However, the predominance of cross-sectional studies limits inference regarding whether muscle loss precedes, follows, or develops in parallel with pulmonary structural changes.

In addition to muscle quantity, CT attenuation provides information on muscle composition, with lower attenuation commonly used as an indicator of greater intramuscular fat infiltration or myosteatosis. Reduced muscle attenuation has been associated with lower exercise capacity and impaired physical performance (48). CT-derived muscle phenotyping therefore includes both quantitative and compositional measures. Comparability across studies remains limited by variation in anatomical landmarks, absolute or normalized muscle indices, attenuation thresholds, and analytical procedures.

### 5.2 Ultrasound

Ultrasound offers a noninvasive and clinically accessible method for assessing respiratory muscle morphology and function, with the diaphragm being the most extensively studied target in COPD. Diaphragmatic abnormalities may reflect dysfunction, adaptation to chronic mechanical loading, or a combination of both, and their interpretation depends on disease state and the parameter measured. During acute exacerbations, ultrasound-detected diaphragmatic impairment has been associated with a higher risk of noninvasive ventilation failure and mortality (49). In stable COPD, impaired diaphragmatic function has been linked to reduced respiratory reserve, poorer pulmonary function, and greater exacerbation risk (50, 51). Measurements of thickness, thickening fraction, excursion, and contractile performance represent distinct physiological domains and should not be treated as interchangeable indicators of respiratory muscle dysfunction.

Ultrasound can also characterize peripheral muscle morphology, particularly the rectus femoris. In COPD, reduced rectus femoris thickness and cross-sectional area have been reported relative to healthy controls and correlate with fat-free mass, quadriceps strength, and exercise capacity (52, 53). These measures support practical bedside phenotyping of peripheral muscle impairment and may complement assessments of muscle strength and exercise capacity. Shear-wave elastography offers additional information on muscle mechanical properties, but its reproducibility, prognostic value, and clinical applicability in COPD require further study (54).

### 5.3 Magnetic resonance imaging

Magnetic resonance imaging (MRI) enables high-resolution characterization of skeletal muscle morphology and respiratory muscle function without ionizing radiation. It permits direct quantification of muscle cross-sectional area and volume and may therefore complement anthropometric and other indirect measures of muscle quantity in COPD (55). MRI-based evaluation of the diaphragm has also identified associations between diaphragmatic abnormalities, disease severity, and exacerbation frequency, indicating possible value for respiratory muscle phenotyping and risk assessment (56). Small study populations and variation in acquisition protocols, anatomical coverage, normalization methods, and measurement procedures currently limit cross-study comparability and broader clinical implementation.

## 6 Shared systemic mechanisms underlying lung–muscle relationships in COPD

**Figure 1:**
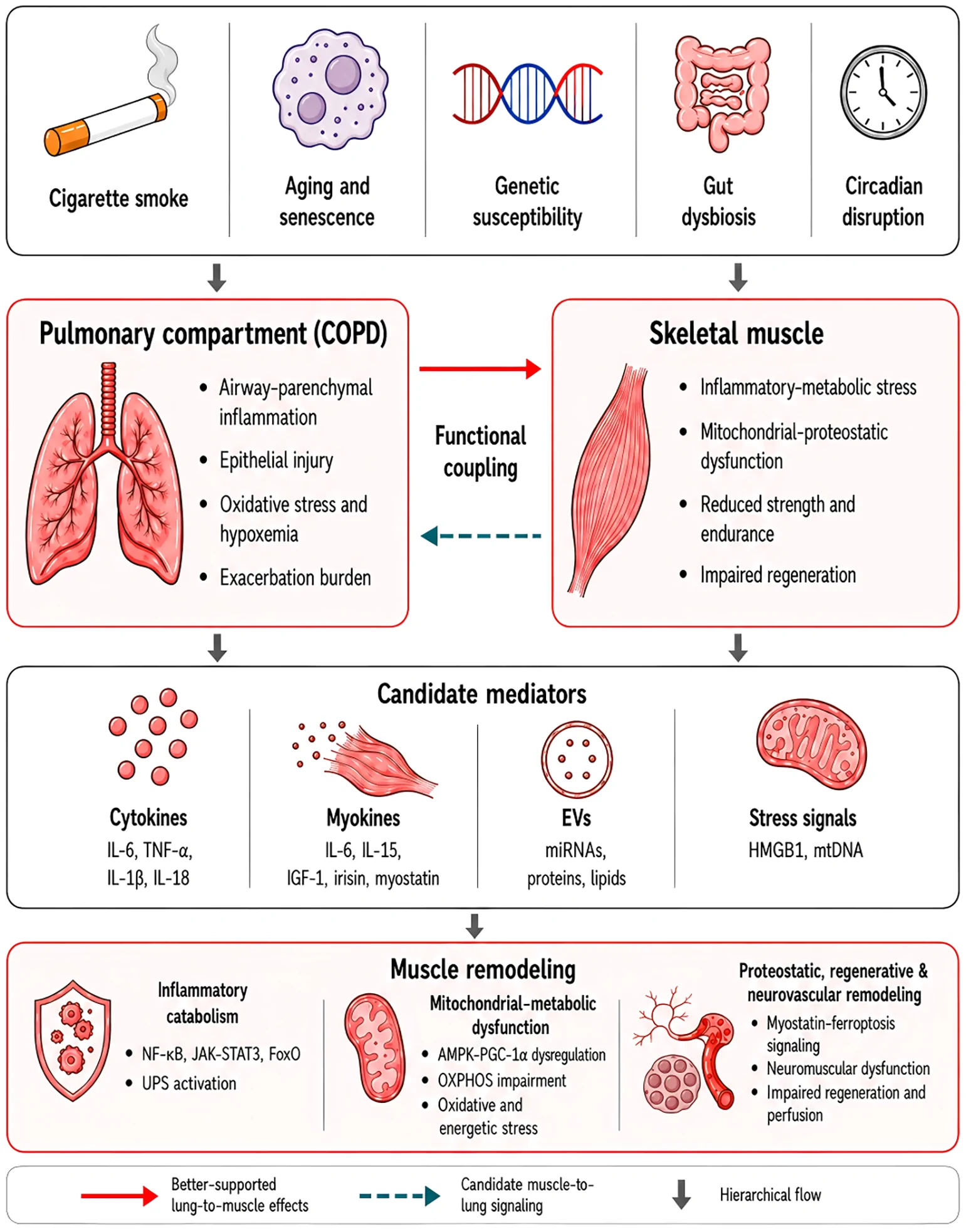
Conceptual framework of lung–muscle relationships in COPD. Shared upstream determinants may contribute to pulmonary and skeletal muscle abnormalities in COPD, with candidate mediators potentially linking these changes to downstream muscle remodeling. Red solid arrows indicate relatively better-supported lung-to-muscle effects, integrating evidence from human clinical studies and COPD-relevant experimental models. Teal dashed arrows indicate emerging muscle-to-lung signaling based predominantly on indirect or hypothesis-generating evidence, whereas gray arrows indicate shared upstream influences and hierarchical flow. Arrow direction and style reflect the relative direction and context of the available evidence and do not imply established causality. The initial figure was created by the authors using Adobe Illustrator 2021 and Adobe generative-AI tools. ChatGPT (GPT-5.5, OpenAI) was subsequently used to assist with refinement of the layout and visual elements. All icons and graphical elements were created or generated specifically for this figure and were not reproduced from previously published figures or third-party copyrighted materials. The scientific content, labels, interpretation, and final design were independently reviewed and approved by the authors. AMPK, AMP-activated protein kinase; COPD, chronic obstructive pulmonary disease; EVs, extracellular vesicles; FoxO, forkhead box O transcription factors; HMGB1, high mobility group box 1; IGF-1, insulin-like growth factor 1; IL, interleukin; JAK, Janus kinase; miRNAs, microRNAs; mtDNA, mitochondrial DNA; NF-κB, nuclear factor kappa B; OXPHOS, oxidative phosphorylation; PGC-1α, peroxisome proliferator-activated receptor gamma coactivator 1-alpha; STAT3, signal transducer and activator of transcription 3; TNF-α, tumor necrosis factor-alpha; UPS, ubiquitin–proteasome system.

Clinical, molecular, and experimental studies suggest that pulmonary and skeletal muscle abnormalities in COPD develop within a shared systemic milieu characterized by chronic inflammation, metabolic stress, impaired tissue repair, and altered cellular adaptation. Immune dysregulation, mitochondrial dysfunction, proteostatic imbalance, and metabolic reprogramming may affect both compartments while producing tissue-specific manifestations. These processes provide a conceptual basis for organizing lung–muscle relationships into shared susceptibility factors, systemic mediators, functional coupling, and inter-organ signaling pathways (**Figure 1**; **Table 4**). For clarity, the mechanistic evidence discussed below is considered at three levels: mechanisms demonstrated in patients with COPD, mechanisms supported by COPD-relevant experimental models, and hypothesis-generating mechanisms extrapolated from other diseases or biological systems.

**Table 4:** Mechanistic pathways in COPD-associated lung–muscle dysfunction.

Evidence level	Mechanism	Main implication	References
Human COPD evidence	Inflammatory–proteostatic remodeling	Associations with muscle loss, weakness, and anabolic–catabolic imbalance	(60, 91, 113–115, 162)
	Mitochondrial–metabolic dysfunction	Reduced energetic efficiency and muscle function	(23, 93, 116, 118)
	Neuromuscular–vascular remodeling	Impaired diaphragm function, perfusion, and exercise adaptation	(121–126)
COPD-model evidence	Smoke-induced muscle catabolism	Proteostatic disruption, atrophy, and weakness	(59, 70, 71)
	Myostatin signaling	Myofiber injury and impaired muscle homeostasis	(114, 115)
Extrapolated/hypothesis-generating evidence	Systemic susceptibility modifiers	Potential modulation of inflammation, metabolism, and muscle vulnerability	(61, 62, 64, 66–68, 74, 75, 77, 78)
	Immune–stromal regulation	Candidate regulation of immune resolution, regeneration, and matrix remodeling	(96–101)
	Candidate inter-organ signaling	Potential systemic signaling; directional muscle-to-lung transfer unconfirmed	(102, 104, 107, 109, 110)

COPD, chronic obstructive pulmonary disease. Evidence levels indicate the directness of mechanistic support in the COPD context rather than causal certainty. Only representative mechanisms are shown.

### 6.1 Shared systemic determinants and biological susceptibility

Genetic, molecular, and systems-level studies suggest that pulmonary and skeletal muscle abnormalities in COPD may partly arise from overlapping biological susceptibility and are unlikely to be explained exclusively by the consequences of advanced respiratory disease. Genetic variants related to muscle performance and metabolic regulation have been associated with interindividual variation in muscle-related phenotypes in COPD (57, 58). Studies of pulmonary and skeletal muscle tissues have also identified alterations in stress-response and metabolic pathways involving sirtuin 1 (SIRT1) and forkhead box O (FoxO) signaling (59, 60). These shared molecular patterns support the presence of parallel cellular adaptations involving stress responses, energy metabolism, and tissue resilience.

Accelerated biological aging may further shape multisystem abnormalities in COPD. Senescence-associated features, including mitochondrial dysfunction, telomere attrition, impaired proteostasis, and inflammatory secretory phenotypes, have been described in both pulmonary and skeletal muscle compartments (61–63). In the lung, senescent epithelial and stromal cells exhibit increased production of mediators such as interleukin-6 (IL-6), interleukin-8 (IL-8), and transforming growth factor-β (TGF-β), which may sustain local inflammation and increase systemic inflammatory burden (64, 65). In skeletal muscle, cellular senescence and impaired regenerative capacity are associated with reduced satellite cell function and greater susceptibility to catabolic remodeling (66–68). Together, these alterations provide a shared aging-related context for pulmonary and skeletal muscle dysfunction.

Cigarette smoke is a major environmental determinant of both pulmonary and skeletal muscle homeostasis. In addition to initiating pulmonary injury, smoke exposure induces systemic oxidative, inflammatory, and metabolic stress that may impair muscle function before severe airflow limitation develops (69). Experimental models show that cigarette smoke disrupts muscle protein homeostasis through reduced protein kinase B (Akt) signaling and FoxO-dependent induction of atrophy-related genes, including muscle atrophy F-box protein (MAFbx/Atrogin-1) and muscle RING finger 1 (MuRF1), thereby increasing ubiquitin–proteasome-mediated protein degradation (70, 71). Reactive constituents such as acrolein further suppress anabolic signaling, increase oxidative stress, and promote myofiber atrophy (72). These mechanistic findings are consistent with clinical observations of impaired muscle function among smokers and patients with COPD.

Alterations in gut microbial composition and intestinal barrier integrity have been associated with inflammatory and muscular abnormalities in COPD-related settings (73–75). In parallel, circadian regulators, including brain and muscle ARNT-like protein 1 (BMAL1) and nuclear receptor subfamily 1 group D member 1 (REV-ERBα), regulate AMP-activated protein kinase (AMPK)–mechanistic target of rapamycin (mTOR) signaling, mitochondrial metabolism, and protein turnover in pulmonary and skeletal muscle tissues (76–78). Through these systemic effects, gut-derived and circadian pathways may modify muscle vulnerability and contribute to heterogeneity in COPD-associated muscle impairment.

### 6.2 Immune–inflammatory and immunometabolic networks

Chronic immune activation is a central component of the systemic pathobiology linking pulmonary disease burden with skeletal muscle dysfunction in COPD. In addition to persistent airway inflammation, COPD involves alterations in innate and adaptive immunity, including changes in circulating neutrophils, monocytes/macrophages, and lymphocytes, together with increased concentrations of inflammatory cytokines, acute-phase proteins, and stress-associated mediators (22, 79–81). Activation of pattern-recognition receptors, macrophage and neutrophil responses, and inflammasome-related pathways may amplify cytokine networks involving interleukin-1β (IL-1β), IL-6, interleukin-18 (IL-18), and tumor necrosis factor-α (TNF-α) (82, 83). Systemic inflammatory activation is associated with reduced muscle strength, impaired exercise capacity, and more rapid functional decline, particularly during acute exacerbations (84). Circulating immune mediators may therefore provide a biological link between pulmonary inflammation and skeletal muscle remodeling.

Within skeletal muscle, inflammatory signaling alters anabolic regulation, protein turnover, metabolic adaptation, and regenerative capacity. Persistent inflammatory activity is associated with disruption of anabolic pathways involving testosterone, insulin-like growth factor-1 (IGF-1), and dehydroepiandrosterone, increasing susceptibility to muscle loss and impaired repair (85). Toll-like receptor and NOD-like receptor family pyrin domain-containing 3 (NLRP3) inflammasome signaling may further intensify IL-1β- and IL-18-related responses in muscle tissue (86, 87). Downstream activation of Janus kinase/signal transducer and activator of transcription 3 (JAK/STAT3) and nuclear factor kappa B (NF-κB) pathways connects cytokine exposure with catabolic and myogenic programs. IL-6–STAT3 signaling can promote FoxO-dependent expression of atrophy-related genes, including MAFbx (Atrogin-1), whereas TNF-α–NF-κB signaling suppresses myogenic differentiation 1 (MyoD)-associated myogenesis (88, 89). These pathways may impair contractile function and mitochondrial adaptation before substantial loss of muscle mass becomes apparent (90). Consistent with these mechanisms, transcriptomic analyses of quadriceps muscle from patients with severe COPD have identified inflammatory gene programs associated with circulating cytokine profiles and reduced muscle mass (91).

Inflammatory activation and metabolic remodeling are closely integrated in skeletal muscle dysfunction in COPD. Chronic inflammatory signaling can alter mitochondrial function, substrate utilization, and energy homeostasis, thereby impairing cellular adaptation (92). Skeletal muscle in COPD exhibits mitochondrial abnormalities, reduced oxidative phosphorylation, increased oxidative stress, and altered substrate use, changes associated with lower energetic efficiency and functional capacity (93). TNF-α-induced NF-κB activation may enhance hypoxia-inducible factor 1-alpha (HIF-1α) signaling, favoring glycolytic reprogramming and suppressing oxidative metabolism (94). Persistent inflammatory stress can also disturb mitochondrial quality control, reactive oxygen species homeostasis, and metabolic flexibility, creating a self-reinforcing cycle of immune activation and metabolic dysfunction (95). Together, these processes increase skeletal muscle vulnerability and impair functional adaptation in COPD.

The local muscle microenvironment adds another layer of control over tissue adaptation and repair. Skeletal muscle contains resident and recruited immune cells that interact with myofibers, satellite cells, fibro-adipogenic progenitors (FAPs), and other stromal populations to shape regenerative and inflammatory responses (96, 97). In experimental muscle injury, they transition from inflammatory states involved in debris clearance toward reparative phenotypes associated with IL-10, IGF-1, satellite cell activation, and tissue remodeling (98, 99). Regulatory T cells also support immune resolution and regenerative responses, while FAPs act as immune-responsive stromal cells that interact with macrophages and myogenic populations to regulate extracellular matrix remodeling (100, 101). In COPD muscle, transcriptomic and molecular studies have identified inflammatory signatures and impaired regenerative programs, suggesting disruption of these immune–stromal networks. Direct characterization of intramuscular immune-cell composition and macrophage phenotypes in COPD remains an important priority.

Muscle stress may also influence systemic immune activity. Injury, accelerated proteolysis, and mitochondrial dysfunction can promote the release of damage-associated molecular patterns (DAMPs), including high-mobility group box 1 (HMGB1) and mitochondrial-derived signals, which activate Toll-like receptor 4 (TLR4)/receptor for advanced glycation end products (RAGE)-dependent NF-κB pathways (102–104). These signals provide a plausible route through which peripheral tissue injury could amplify systemic inflammation. Their specific contribution to pulmonary immune responses in COPD, however, remains a hypothesis requiring direct functional investigation.

Taken together, systemic immune activation, cytokine-mediated signaling, and local immune–stromal interactions may represent an important component of the mechanisms underlying skeletal muscle dysfunction in COPD. Through their effects on protein turnover, metabolic adaptation, regenerative capacity, and tissue homeostasis, immune processes may contribute to the development and persistence of muscle impairment.

### 6.3 Inter-organ communication networks

Circulating myokines and extracellular vesicles (EVs) provide potential routes for molecular communication within the lung–muscle framework. Myokines represent soluble endocrine signals released by skeletal muscle, whereas EVs can transport proteins, lipids, and regulatory RNAs through the circulation, thereby extending intercellular signaling across tissues.

Skeletal muscle functions as an endocrine organ through the secretion of myokines with autocrine, paracrine, and endocrine effects on muscle homeostasis and systemic physiology (105). In COPD, altered myokine profiles have been associated with impaired muscle adaptation, metabolic dysregulation, and reduced regenerative capacity (20). Several myokines and muscle-regulatory mediators, including IL-6, interleukin-15 (IL-15), IGF-1, irisin, and myostatin, have been implicated in COPD-associated muscle dysfunction (106). IL-6 has context-dependent effects: transient release from contracting muscle participates in metabolic adaptation and tissue remodeling, whereas persistently elevated circulating IL-6 in COPD is associated with systemic inflammation, reduced muscle strength, impaired exercise capacity, and greater disease severity (107). Myokine signaling may therefore connect muscle activity and metabolic status with broader inflammatory and adaptive responses. Exercise can modify circulating myokine profiles, although the magnitude and direction of these changes vary with disease severity, hypoxemia, and individual characteristics (108).

EVs provide an additional mode of circulating molecular signaling. Their cargo includes proteins, lipids, and non-coding RNAs capable of modifying metabolic, inflammatory, and stress-response pathways in recipient cells. Altered EV abundance and regulatory RNA profiles have been reported in COPD, linking vesicular signaling with disease-associated inflammatory and metabolic changes (109). Circulating EVs containing muscle-enriched microRNAs, including miR-206 and members of the miR-133 family, have also been detected in patients with COPD, consistent with a contribution of skeletal muscle to the circulating EV pool (110). Exercise-induced release of skeletal muscle-derived EVs further indicates that muscle activity can influence systemic signaling through vesicular cargo (111). However, the directional transfer of defined muscle-derived EVs to pulmonary tissues and their functional effects in COPD have not yet been established.

Together, myokines and EVs may provide complementary routes through which changes in skeletal muscle activity, metabolism, and tissue stress are conveyed to the systemic circulation. Myokines act as soluble endocrine mediators, whereas EVs transport complex molecular cargo that may influence inflammatory and metabolic responses in COPD.

### 6.4 Tissue-level dysfunction and remodeling

At the tissue level, skeletal muscle dysfunction in COPD reflects the cumulative disruption of cellular maintenance, energy metabolism, regenerative processes, and structural integrity. These changes translate systemic and local stress into reduced contractile performance, impaired recovery, and diminished adaptability.

Loss of proteostatic balance is a prominent feature of COPD-associated muscle wasting. Activation of proteolytic pathways, particularly the ubiquitin–proteasome system, is accompanied by increased expression of the muscle-specific E3 ubiquitin ligases MAFbx (Atrogin-1) and MuRF1 in respiratory and peripheral muscles (112, 113). Myostatin-related signaling and ferroptotic pathways have also been implicated in COPD-associated muscle remodeling (114, 115). Disruption of the balance among protein degradation, cellular renewal, and tissue repair progressively compromises muscle quality and regenerative potential.

Metabolic dysfunction further limits tissue adaptation. Skeletal muscle in COPD commonly exhibits reduced oxidative capacity, mitochondrial abnormalities, and altered substrate utilization, leading to lower energetic efficiency during exercise and other functional demands (23, 116). Changes in peroxisome proliferator-activated receptor gamma coactivator 1-alpha (PGC-1α)-dependent mitochondrial biogenesis, AMPK-mediated energy sensing, and hypoxia-responsive pathways may reduce metabolic flexibility and increase susceptibility to fatigue and functional decline (117, 118).

COPD-associated respiratory mechanics and neuromuscular abnormalities may impair diaphragmatic activation and respiratory performance (119, 120). Altered microRNA-1 (miR-1)-related signaling has also been reported in the quadriceps of patients with COPD and may contribute to neuromuscular remodeling (121). In parallel, endothelial dysfunction, impaired vascular endothelial growth factor (VEGF)-related angiogenic signaling, capillary rarefaction, and reduced nitric oxide bioavailability may restrict muscle perfusion and oxygen delivery, thereby limiting exercise adaptation and muscle function (122–126).

The resulting muscle phenotype varies across anatomical and functional compartments. Differences in mechanical loading, fiber composition, metabolic demand, vascular supply, and regenerative reserve may help explain why peripheral weakness, reduced endurance, muscle wasting, and respiratory muscle dysfunction do not develop uniformly in COPD. Longitudinal and tissue-resolved studies could further define the mechanisms underlying these distinct patterns.

## 7 Therapeutic strategies targeting COPD-associated muscle dysfunction

Interventional studies indicate that skeletal and respiratory muscle dysfunction are modifiable components of COPD. Available strategies include exercise training, respiratory muscle conditioning, nutritional and metabolic support, pharmacological approaches, and multimodal rehabilitation. Their benefits are most consistently observed in muscle strength, exercise capacity, dyspnea, physical function, and health-related quality of life, although responses vary according to disease severity, nutritional status, baseline muscle impairment, and intervention design (**Table 5**).

**Table 5:** Therapeutic strategies for COPD-associated muscle dysfunction.

Strategy	Outcomes	Evidence base	References
Exercise-based rehabilitation	Strength, exercise capacity, physical function, and quality of life; variable muscle-mass and aerobic responses	Consensus statements; RCTs; meta-analyses	(4, 127–132)
Respiratory muscle training	Inspiratory strength; variable effects on dyspnea, exercise capacity, and quality of life	RCTs; systematic reviews	(133–135)
Nutritional and metabolic modulation	Nutritional status, physical performance, and endurance; heterogeneous muscle-function responses	RCTs; clinical and translational studies	(136–141)
Pharmacological and molecular approaches	Fat-free mass and proteolytic, redox, and mitochondrial pathways; inconsistent functional gains	RCT; cell-based, preclinical, and translational studies	(141–144)
Neuromuscular electrical stimulation	Functional exercise capacity in severe or exercise-limited COPD	Pilot and placebo-controlled RCTs	(146, 147)
Nutritional support plus rehabilitation	Lean-mass preservation and quadriceps strength	RCT; clinical review	(148, 149)

COPD, chronic obstructive pulmonary disease; RCTs, randomized controlled trials.

### 7.1 Exercise-based interventions

Exercise training is a central component of the management of COPD-associated muscle dysfunction. Pulmonary rehabilitation programs incorporating endurance and resistance exercise improve peripheral muscle strength, exercise capacity, and health-related quality of life, whereas changes in muscle mass vary according to disease severity, nutritional status, and training characteristics (4, 127).

Randomized controlled trials have shown that combined endurance and resistance training can increase quadriceps strength, aerobic capacity, and exercise tolerance, while also inducing imaging-detectable changes in muscle structure (128, 129). Concurrent programs incorporating power-based exercise and high-intensity interval components have also improved physical function and muscle power, with some preservation of muscle thickness after training cessation, although effects on maximal aerobic capacity have been inconsistent (130). Meta-analyses similarly report improvements in quadriceps strength and walking performance after resistance training, with substantial variation across protocols and patient populations (131). High-intensity interval training (HIIT) offers an alternative training modality and may improve exercise capacity, symptoms, and peripheral muscle metabolic adaptation in selected patients (132).

Overall, exercise-based interventions improve multiple dimensions of muscle and physical function in COPD, although gains in muscle mass, aerobic capacity, and long-term maintenance are not uniform across individuals.

### 7.2 Respiratory muscle training

Respiratory muscle impairment is a distinct component of COPD-associated muscle dysfunction, particularly in patients exposed to increased ventilatory loading and altered respiratory mechanics. Airflow limitation, lung hyperinflation, and elevated respiratory workload increase the demands placed on inspiratory muscles and may reduce respiratory muscle reserve.

Respiratory muscle training (RMT), particularly inspiratory muscle training (IMT), can improve inspiratory muscle strength and endurance in COPD and may also benefit selected measures of dyspnea and exercise capacity, although effects on quality of life are less consistent (133). Individual trials have reported additional improvements when IMT is combined with pulmonary rehabilitation (134). However, broader evidence indicates that adjunctive IMT improves inspiratory muscle strength without consistent additional benefits in dyspnea, functional exercise capacity, or health-related quality of life compared with pulmonary rehabilitation alone (135). These findings highlight the distinction between improvements in respiratory muscle performance and broader clinical outcomes.

### 7.3 Metabolic and nutritional modulation

Nutritional and metabolic interventions aim to address systemic factors associated with muscle depletion and impaired physical function in COPD. Leucine-enriched formulations, vitamin D, and preparations containing polyunsaturated fatty acids have been studied in patients with COPD and muscle wasting. Reported benefits include improvements in nutritional status, body-weight maintenance, physical activity, and selected health-related outcomes, whereas effects on muscle strength and exercise capacity have varied (136–138). Gut-derived metabolites and micronutrient deficiencies may also influence muscle function. In a randomized clinical trial, butyrate supplementation improved handgrip strength and physical performance and reduced markers associated with intestinal permeability and neuromuscular junction degradation (139). Correction of iron deficiency has likewise been associated with improved endurance capacity and disease-related symptoms in selected patients with COPD (140). Metabolic modulation with dichloroacetate, an activator of pyruvate dehydrogenase, reduced exercise-induced lactate accumulation and improved exercise performance, although it remains investigational (141). Collectively, these studies suggest that nutritional status, intestinal metabolic pathways, and substrate utilization may represent modifiable influences on COPD-associated muscle impairment.

### 7.4 Pharmacological and molecular interventions

Pharmacological approaches targeting muscle anabolism, proteolysis, oxidative stress, and mitochondrial regulation have also been investigated, but few have progressed into routine clinical use.

Nandrolone decanoate increased fat-free mass in patients with COPD undergoing pulmonary rehabilitation, while effects on muscle function and exercise capacity were inconsistent (142). This dissociation indicates that increasing muscle mass alone may not restore functional performance. In COPD-derived myotubes, roflumilast-induced cyclic adenosine monophosphate (cAMP) signaling reduced proteolytic activity and modified antioxidant and myogenic pathways, while animal studies suggest that modulation of oxidative stress and muscle regulatory pathways can attenuate cigarette smoke-induced muscle atrophy (143, 144). Dickkopf-related protein 3 (DKK3) has also been proposed as a molecular regulator associated with mitochondrial dysfunction and muscle atrophy in COPD-associated sarcopenia (145). These findings identify candidate therapeutic pathways, although their clinical efficacy and safety require evaluation in appropriately designed intervention studies.

### 7.5 Combined rehabilitation strategies

Multimodal rehabilitation combines interventions that address different dimensions of COPD-associated muscle impairment. Neuromuscular electrical stimulation (NMES) provides an alternative or adjunctive approach for patients with severe respiratory impairment or limited capacity to participate in conventional exercise training. Randomized controlled trials of respiratory and peripheral muscle NMES, including quadriceps stimulation, have reported improvements in functional exercise capacity, particularly 6MWD, with favorable safety profiles in patients with severe COPD (146, 147). Adjunctive nutritional support during rehabilitation has been associated with preservation of lean body mass and improved quadriceps strength, while broader clinical reviews support integrated nutritional and rehabilitative management in COPD (148, 149). By addressing muscle activation, nutritional status, and exercise tolerance concurrently, multimodal programs may offer a complementary approach to the management of COPD-associated muscle impairment.

### 7.6 Clinical translation

Recognition of COPD-associated muscle impairment supports a multidimensional assessment that complements conventional pulmonary function testing. Clinical evaluation may include measures of muscle strength and performance, such as handgrip strength and quadriceps function; assessments of exercise capacity, including the 6MWD and 1STST; imaging-derived measures of peripheral and respiratory muscle morphology and function; and evaluation of nutritional status and exacerbation history. Together, these measures can characterize different dimensions of muscle impairment and support functional assessment, risk stratification, and individualized rehabilitation planning. Longitudinal studies integrating clinical phenotyping with inflammatory, metabolic, and molecular profiling could help identify distinct muscle-related phenotypes and determine which patients are most likely to benefit from specific interventions.

## 8 Discussion

Skeletal muscle dysfunction is an integral extrapulmonary component of COPD and cannot be explained solely as a secondary consequence of advanced airflow limitation. Findings from epidemiological studies, longitudinal cohorts, imaging investigations, genetic analyses, molecular studies, and experimental models collectively indicate that pulmonary disease burden and muscle impairment are connected through shared susceptibility factors, systemic biological disturbances, biomechanical constraints, and functional coupling. Clinically, these relationships are reflected in reduced exercise capacity, impaired physical function, greater exacerbation risk, and adverse outcomes.

The biological processes involved are multidimensional. Chronic inflammation, oxidative stress, mitochondrial dysfunction, impaired proteostasis, cellular aging, vascular alterations, neuromuscular remodeling, and metabolic disturbances can affect pulmonary and skeletal muscle homeostasis through overlapping pathways (10, 18). Circulating cytokines, endocrine mediators, and changes in substrate metabolism provide systemic links between the two compartments, while myokines and extracellular vesicles represent candidate routes for inter-organ signaling (150–152). The lung–muscle framework therefore accommodates shared systemic mechanisms, tissue-specific responses, functional coupling, and molecular communication without reducing these relationships to a single linear pathway.

The manifestations of muscle impairment vary according to COPD phenotype, disease stage, environmental exposure, and host susceptibility (4). In emphysema-predominant disease, hyperinflation, altered respiratory mechanics, and increased ventilatory workload may place greater demands on the diaphragm and other respiratory muscles (153). Chronic bronchitis-predominant disease may be accompanied by persistent inflammatory activity and recurrent exacerbations that adversely affect peripheral muscle homeostasis (154). Chronic hypoxemia, physical inactivity, nutritional imbalance, aging-related anabolic resistance, and metabolic abnormalities may further influence muscle function through altered oxygen delivery, mitochondrial stress, and reduced regenerative capacity (155–158). These factors may also affect respiratory and peripheral muscles differently because of variation in mechanical loading, fiber composition, metabolic demand, and vascular supply.

Lung–muscle relationships may begin before established COPD. Individuals with smoking-related structural abnormalities, early airflow impairment, or accelerated lung function decline may also exhibit reduced physical activity, systemic inflammation, and metabolic alterations associated with muscle vulnerability (69, 159). Cigarette smoke can affect pulmonary and skeletal muscle tissues through oxidative stress, inflammatory activation, and mitochondrial injury (160, 161). Biomass exposure may produce different pulmonary phenotypes while engaging partially overlapping inflammatory, hypoxic, and metabolic pathways. Greater representation of diverse exposure patterns and patient populations will be necessary to determine how these factors shape pulmonary and muscular trajectories.

From a clinical perspective, the available findings support assessment of muscle strength, physical performance, respiratory muscle function, nutritional status, and imaging-derived muscle characteristics alongside conventional pulmonary measures. Exercise training, pulmonary rehabilitation, respiratory muscle training, nutritional support, and multimodal rehabilitation can improve selected functional outcomes, although responses vary across muscle phenotypes and patient characteristics. The dissociation between muscle mass and muscle function further indicates that therapeutic evaluation should incorporate strength, endurance, exercise capacity, and patient-centered outcomes rather than relying on a single structural measure.

## 9 Evidence gaps and future perspectives

Several limitations constrain interpretation of the current literature. Definitions and assessment methods vary across sarcopenia, muscle wasting, weakness, and impaired physical performance, while respiratory and peripheral muscles differ in structure, loading, and adaptive responses. Most clinical studies are observational or cross-sectional, limiting causal inference and leaving the direction of lung–muscle relationships uncertain. Age, smoking, physical inactivity, corticosteroid exposure, malnutrition, comorbidities, and disease severity may affect both pulmonary and muscle outcomes, and findings obtained during stable COPD may not apply to acute exacerbations. Genetic studies also rely largely on selected quantitative traits and predominantly European-ancestry populations, which may restrict generalizability.

Mechanistic interpretation is similarly challenging. The tissue sources of circulating cytokines, myokines, microRNAs, and extracellular vesicles are often unclear, making it difficult to distinguish causal mediators from markers of systemic disease burden. Direct evidence for muscle-to-lung signaling remains scarce, and findings from non-COPD experimental models may not fully reflect COPD biology. Results are also inconsistent across muscle traits, imaging measures, genetic analyses, and interventions; improvements in muscle mass, for example, do not consistently translate into greater strength or exercise capacity. Publication bias and citation selection may further favor supportive findings. Future longitudinal, tissue-resolved, and intervention-based studies in diverse populations are needed to define temporal relationships, identify causal pathways, and determine the clinical relevance of distinct muscle phenotypes.

## 10 Conclusion

The lung–muscle framework positions skeletal muscle dysfunction as an integral component of COPD heterogeneity and clinical burden. Clinical findings support associations among pulmonary impairment, muscle dysfunction, reduced physical capacity, and adverse outcomes. Assessment of muscle strength, physical performance, respiratory muscle function, nutritional status, and imaging-derived muscle characteristics may therefore complement conventional pulmonary evaluation and inform rehabilitation planning. Shared inflammatory, metabolic, mitochondrial, vascular, and aging-related processes provide plausible biological links between pulmonary and skeletal muscle abnormalities, while the direction and tissue specificity of molecular signaling remain to be defined. Longitudinal, tissue-resolved, and interventional studies are needed to identify causal mechanisms and determine whether their modification improves clinically meaningful outcomes.

## Glossary

1STST: 1-minute sit-to-stand test
6MWD: six-minute walk distance
ALM: appendicular lean mass
AMPK: AMP-activated protein kinase
Akt: protein kinase B
BMAL1: brain and muscle aryl hydrocarbon receptor nuclear translocator-like protein 1
cAMP: cyclic adenosine monophosphate
COPD: chronic obstructive pulmonary disease
CT: computed tomography
DAMPs: damage-associated molecular patterns
DKK3: Dickkopf-related protein 3
EVs: extracellular vesicles
FAPs: fibro-adipogenic progenitors
FEV₁: forced expiratory volume in one second
FoxO: forkhead box O transcription factors
FVC: forced vital capacity
HGS: handgrip strength
HIF-1α: hypoxia-inducible factor 1-alpha
HIIT: high-intensity interval training
HMGB1: high mobility group box 1
IGF-1: insulin-like growth factor 1
IL: interleukin
IMT: inspiratory muscle training
JAK: Janus kinase
MAFbx: muscle atrophy F-box protein
miRNAs: microRNAs
MR: Mendelian randomization
MRI: magnetic resonance imaging
mtDNA: mitochondrial DNA
mTOR: mechanistic target of rapamycin
MuRF1: muscle RING finger 1
MyoD: myogenic differentiation 1
NF-κB: nuclear factor kappa B
NLRP3: NOD-like receptor family pyrin domain-containing 3
NMES: neuromuscular electrical stimulation
OXPHOS: oxidative phosphorylation
PGC-1α: peroxisome proliferator-activated receptor gamma coactivator 1-alpha
RAGE: receptor for advanced glycation end products
RCT: randomized controlled trial
REV-ERBα: nuclear receptor subfamily 1 group D member 1
RMT: respiratory muscle training
SIRT1: sirtuin 1
SMD: skeletal muscle dysfunction
STAT3: signal transducer and activator of transcription 3
TGF-β: transforming growth factor beta
TLR4: Toll-like receptor 4
TNF-α: tumor necrosis factor alpha
UPS: ubiquitin–proteasome system
VEGF: vascular endothelial growth factor.
